# Origin of the pH Dependency of EPR Parameters: The Case of a Protonatable Nitroxide in Aqueous Solution

**DOI:** 10.1021/acs.jpclett.5c01053

**Published:** 2025-08-01

**Authors:** Laura Galazzo, Stefan Maste, Bikramjit Sharma, Van Anh Tran, Tim Pongratz, Markus Teucher, Dominik Marx, Frank Neese, Stefan M Kast, Enrica Bordignon

**Affiliations:** † Department of Physical Chemistry, 34326 University of Geneva, 30 Quai Ernest Ansermet, 1211 Geneva, Switzerland; ‡ Fakultät für Chemie und Chemische Biologie, 14311 Technische Universität Dortmund, 44227 Dortmund, Germany; § Lehrstuhl für Theoretische Chemie, 9142 Ruhr-Universität Bochum, 44780 Bochum, Germany; ∥ 28314 Max-Planck-Institut für Kohlenforschung, 45470 Mülheim an der Ruhr, Germany; ⊥ Max-Planck-Institut für Chemische Energiekonversion, 45470 Mülheim an der Ruhr, Germany

## Abstract

Protonatable nitroxides are electron paramagnetic resonance (EPR) molecular probes employed for pH measurements in bulk aqueous media. The change in the protonation state of the molecule induces a measurable change in the *g*- and hyperfine (*A*) parameters used as pH indicators. The quantitative understanding of the origin of the change of the EPR parameters in terms of electronic structure and different solvation patterns is still lacking. Here, we delve into the origins of the changes in the *g*- and hyperfine (*A*) parameters of ^14^N upon protonation of the heterocyclic nitrogen of the pH-sensitive nitroxide probe HMI (2,2,3,4,5,5-hexamethylimidazolidin-1-oxyl, C_9_H_19_N_2_O) by means of combined experimental and theoretical techniques that have been developed and extensively validated in previous works. To establish a molecular-level understanding of the dependency of EPR parameters on the pH of the medium, we considered two limiting cases of deprotonated (pH ≫ p*K*_a_) and protonated (pH ≪ p*K*_a_) states of HMI. We found that, upon protonation of the heterocyclic nitrogen, the change in the electronic structure dominates the pH dependency of isotropic *g* and *A* values. Supporting this prominent role of electronic structure modulation, the average shift of EPR observables between the corresponding hydrogen-bonding states of the protonated and unprotonated forms remains constant. Furthermore, the results establish that the hydrogen bonding network structures around the nucleus of interest only marginally change upon protonation, although the populations of corresponding states with given H-bond numbers strongly do. This feature entails an additional, smaller contribution to the relative pH dependency of *g* ^iso^ and *A* ^iso^ values over electronic structure modulation upon protonation in a given H-bond state. The findings of this study pave the way to investigating HMI-based labels in peptides and other pH-sensitive EPR probes in protic polar solvents.

## Graphical abstract

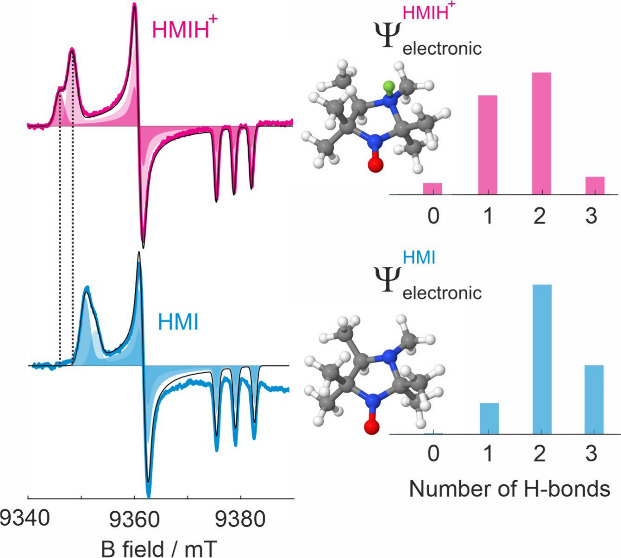

In Electron Paramagnetic Resonance (EPR) studies, nitroxides serve multifaceted roles, ranging from spin labels for probing biomolecular structure and dynamics^1–3^ to paramagnetic probes for investigating chemical reactions,^4^ redox processes,^5^ and material properties.^6^ Their chemical stability, combined with tunable properties such as spin relaxation times and solubility, make them invaluable assets in elucidating intricate biological pathways, characterizing complex materials, and unraveling fundamental chemical phenomena.

The success of nitroxides in elucidating molecular structures and dynamics hinges on their sensitivity to the environmental factors experienced by the electron spin.^2,3^ This sensitivity allows for the characterization of subtle changes in the local environment, such as alterations in pH, which can significantly impact the magnetic properties of the spin labels.^7,8^

Investigating the pH dependency of magnetic tensors provides valuable insights into the protonation equilibria, structural rearrangements, and dynamic processes occurring in biomolecular systems.^9–11^ Moreover, understanding how pH influences the magnetic properties of spin labels is essential for the accurate interpretation of EPR spectra and the reliable extraction of structural and dynamic information, which may have broad applications in *in situ* EPR studies.^12–14^

We had previously reported^15^ an in-depth investigation of the unprotonated form of HMI (2,2,3,4,5,5-hexamethylimidazolidin-1-oxyl, C_9_H_19_N_2_O) (Figure 1) in aqueous solution and showed how the combination of experiment and extensively validated theory is able to differentiate sub-ensembles of HMI with one to three hydrogen bonds to the nitroxy oxygen. We found that the neutral HMI in aqueous solution has a solvation pattern dominated by 2 and 3 H-bonds with distinct *g*_*xx*_ parameters, which allowed the interpretation of the two main spectral components experimentally detected in continuous-wave high-field EPR spectra. Additionally, we applied the methodology to an in-depth investigation of solvation effects on the *g*-tensor anisotropy and strain.^16^ In this work, we extend our focus to the protonated form of HMI (referred to as HMIH^+^) in water at ambient conditions. The aim is to understand the origin of the pH dependence of the hyperfine coupling constant *A* and of the *g*-tensor by comparing the differences in the electronic structure and solvation patterns between the protonated and deprotonated forms of the nitroxide. Using state-of-the-art experimental and computational techniques, we found that the observed pH dependency is governed by two factors: (1) the changes in electronic structure in a specific H-bonding state upon protonation and (2) the modulation of the populations of H-bonding states of the probe as a function of pH that are found to be structurally similar, though occurring with different probabilities for HMI and HMIH^+^. This work paves the way toward more complex theoretical endeavors which could describe other pH-sensitive nitroxides in polar protic solvents or HMI-based labels attached to short peptides or proteins in solutions.

**Figure 1:**
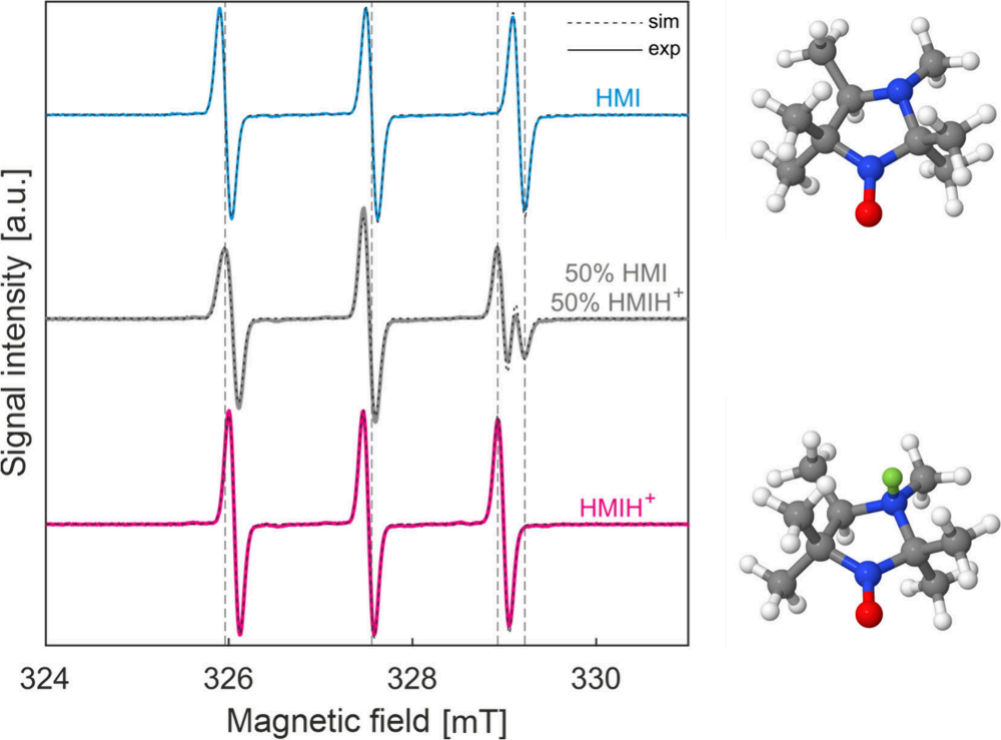
**Room-temperature CW X-band EPR spectra of HMI in water solutions at different pH.** On the left: experimental (blue and magenta, solid line) and EasySpin simulated (black, dashed line) X-band EPR spectra at 298 K for HMI (blue, pH 10) and HMIH^+^ (magenta, pH 2). In gray, the spectrum obtained at pH 4.5 containing both species in equilibrium. Vertical dashed lines highlight the spectral shifts due to changes in both *A* ^iso^ and *g* ^iso^ for the two species. On the right: structures of HMI (top) and HMIH^+^ (bottom) in the 3*R* and (3*R*,4*S*) configurations as used in simulations. The protonation at the nitrogen atom of the ring is highlighted in green.

First, CW-EPR experiments were performed on aqueous solutions of HMIH^+^ and HMI to extract the experimental *A* ^iso^ (to ^14^N) and *g* ^iso^ values at 298 K. We initially prepared solutions of the spin label HMI (synthesized from 2,2,4,5,5-pentamethylimidazolin-1-oxyl, see refs 15,17 ) at pH 2 and pH 10, in order to achieve full protonation and deprotonation, respectively. In addition, a sample was prepared at pH 4.5 to obtain equilibrium conditions in which both states are simultaneously present (Figure 1). From the room-temperature CW-EPR experiments at X band (9.5 GHz) *A* ^iso^ and *g* ^iso^ values were extracted using the MATLAB toolbox Easyspin^18^ (Figure 1). The obtained parameters are *A* ^iso, 298 K^ = 44.87 ± 0.14 MHz and *g* ^iso, 298 K^ = 2.00550 ± 10^–5^ for HMI (taken from^15^) and *A* ^iso, 298 K^ = 41.20 ± 0.14 MHz and *g* ^iso, 298 K^ = 2.00575 ± 10^–5^ for HMIH^+^. These simulations were performed using the “*garlic”* routine in EasySpin and a line width of 0.12 mT, as in our previous work.^15^ The *A* ^iso, 298 K^ values are in line with the ones previously reported^15,16^ for the same nitroxide probe at room temperature. It was also confirmed that, at intermediate pH values, when both species are simultaneously present, the spectral parameters are unchanged, as expected for diluted systems.^19^

To understand the origin of the different EPR parameters in the protonated and deprotonated forms of HMI, we used the protocol previously established.^15^ As two different diastereomers of HMIH^+^ can potentially coexist upon protonation, we calculated the Gibbs energy differences between them using an indirect approach. First, the solvation free energies were calculated with EC-RISM^20,21^ (embedded cluster reference interaction site model) and the revPBE0-D3 functional with the def2-TZVPP basis set with decontracted s-functions on CPCM-optimized structures. Second, the differences of gas phase energies were predicted after optimization *in vacuo* at the same level of theory followed by single-point DLPNO–CCSD­(T)^22–27^ calculations with the same basis set, and added to the solvation free energies. On this level, the (3*R*,4*S*) configuration is favored by 3.54 kcal mol^–1^ (using the partial molar volume correction taken from^21^ 3.08 kcal mol^–1^ without correction) compared to the (3*R*,4*R*) configuration, which leads to more than 99.7% of the population at 298 K for this isomer. Hence, only the (3*R*,4*S*) diastereomer of HMIH^+^ was used in the AIMD simulations. For quantum chemical calculations of *A* ^iso^ and *g* ^iso^ of the ^14^N nucleus, in the same way as previously reported,^15^ vertically desolvated (VD) structures were generated by stripping off all water molecules while preserving the structure of the solute, HMI or HMIH^+^, as found in the aqueous solution. In addition to our earlier investigations, these were also studied *in vacuo* to isolate the electronic structure effect without the inclusion of any solvent. To study the quantitative impact of solvation beyond intramolecular structural perturbation implicitly accounted for by the VD ensemble, a hierarchy of models was employed: explicitly retaining up to the second solvation shell of water molecules around the nitroxy oxygen (SSS) and applying two hybrid solvation models by using either EC-RISM-derived^20,21^ or force field-based background charges (the QM/MM approach outlined in^15^) on top of SSS snapshots, employing the computational setups developed and extensively validated previously.^15,16^ These models are further analyzed on the level of individual sub-ensembles defined by the number of H-bonds. This allows us to investigate the impact of H-bond population changes between HMI and HMIH^+^ in relation to the result of electronic structure modulation in a given H-bonding state.

Quantum chemical calculations of *A* ^iso^ and *g* ^iso^ again employing the revPBE0-D3 functional and the def2-TZVPP basis set with decontracted s-functions were performed for these ensembles, VD, SSS, EC-RISM and QM/MM. All quantum chemical calculations were performed using the ORCA version 5.0.3 ^28^ program package. As demonstrated earlier,^15,16^ a purely DFT-based treatment correctly reproduces trends when it comes to a comparison of various H-bond patterns, though an absolute error remains compared to experiment which can be recovered by referring to much more costly DLPNO–CCSD calculations within the QM/MM scheme^15^ that were not necessary for this study.

The solvation properties of HMI and HMIH^+^ were first compared on the basis of the respective AIMD trajectories. The radial distribution function of the nitroxy-oxygen and water-oxygens (Figure 2a) of HMIH^+^ shows a smaller density of water molecules in the first solvation shell of the nitroxy-oxygen site compared to that of HMI. This is expected as the positive charge of HMIH^+^ reduces the charge density on O, thus diminishing its capability to attract water molecules. To understand the protonation-induced solvation alterations more precisely, we compared the hydrogen-bonding (H-bonding) properties of the O atom of HMI and HMIH^+^ using the same geometric criterion (see caption to Figure 2) as in our earlier study^25^ to define H-bonds and split the AIMD trajectories into subensembles based on the number of water-O H-bond numbers. The quantitative differences in solvation between HMI and HMIH^+^ are listed in Table 1 and depicted in Figure 2. The pair distribution functions between the nitroxy oxygen of HMI/HMIH^+^ and water oxygen shown as inset announce a key insight of this study, namely that the geometric H-bond structure (as reflected by peak maxima and minima locations) is marginally influenced by protonation whereas H-bond populations (reflected by peak heights) differ, as supported by the H-bond analysis provided in Table 1. Corresponding to the reduced O charge density, protonation leads to *decreasing* average H-bond numbers (see Table 1), accompanied by a shift of the sub-ensemble populations favoring less H-bonds.

**Figure 2:**
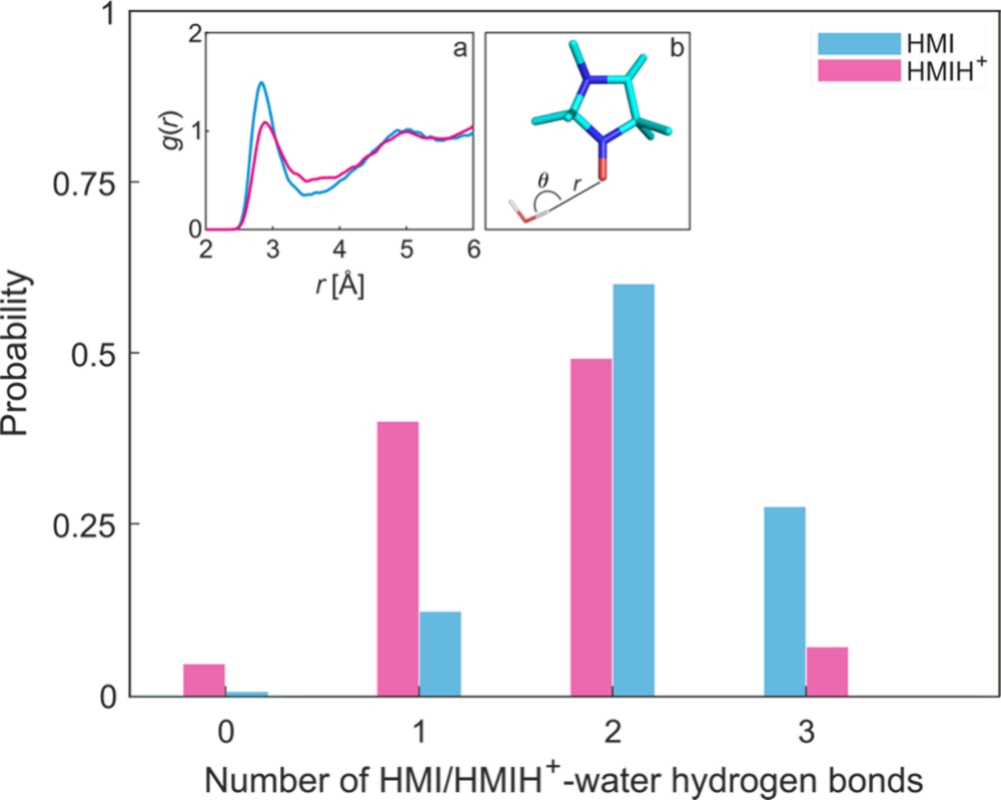
**Probability distribution functions of the number of HMI/HMIH** ^**+**^ **–water H-bonds obtained from AIMD simulations.** The insets show the radial distribution functions *g*(*r*) of the nitroxy-oxygen of HMI/HMIH^+^ and water-oxygens (a) and the geometric variables *r* and θ involved in the definition of H-bond (b). We used the H-bond criterion *r*_O···H_ < 1.71 cos θ + 1.37 as previously established in^15^ for HMI, which we found also applicable to HMIH^+^–water H-bonds.

**Table 1:** Comparison of H-Bond Statistics for HMI and HMIH^+^ in Water^a^

Property	HMIH^+^	HMI^16^
CN	2.70	2.80
⟨*n*_*HB*_⟩	1.58	2.16
*P*_0_	4.4%	0.3%
*P*_1_	**40.0%**	12.0%
*P*_2_	**49.0%**	**59.8%**
*P*_3_	6.6%	**27.2%**
*P*_>3_	0.0%	0.7%

a CN, ⟨*n*_*HB*_⟩, and *P*_*n*_ are coordination number, average number, and population with *n* H-bonds between the nitroxy-oxygen and the water-oxygens, respectively. The populations of the two dominant fractions for each system are highlighted in bold: the 1 and 2 H-bond populations for HMIH^+^ represent 89% of the cases; the 2 and 3 H-bond populations for HMI represent 87% of the cases.

It was previously demonstrated that two major contributions of H-bonding for HMI were experimentally distinguishable in the W- and J-band CW EPR spectra of frozen solutions of HMI (pH 10) in the presence of 10% v/v glycerol as cryoprotectant and could be theoretically assigned to fractions with 2 and 3 H-bonds (namely *P*_2_ and *P*_3_, see Figure 3a).^16^ With respect to HMI, the experimental J-band CW EPR spectrum of HMIH^+^ in frozen solution presented in Figure 3a shows a distinct overall shift of the *g*_*xx*_ region toward lower fields (higher *g*_*xx*_-values). Two major *g*_*xx*_ components are also resolved in this case but, in contrast to HMI, the highest intensity is detected for the peak at higher *B* field, corresponding to lower *g*_*xx*_ - therefore to a higher number of H-bonds (Figure 3). According to the theoretical calculations (Table 1 and simulated spectra in Figure 3a,b), there are two most prominent populations of HMIH^+^ with one H-bond (40% in Table 1) and two H-bonds (49% in Table 1). Using only these two dominant populations (with relative weights renormalized as in Table 2) to simulate the spectra in Figure 3b,c, we could reproduce the features of the experimental spectra of HMIH^+^ with respect to HMI, namely the overall shift of the *g*_*xx*_ region of HMIH^+^ toward lower fields (higher *g*_*xx*_ values) and the redistribution of the two main spectral fractions. As previously found for HMI, the difference between the calculated *g*_*xx*_ values of the two H-bonded populations of HMIH^+^ is slightly smaller than what experimentally observed, which results in the absence of a clear separation between the two spectral components in the theoretical spectra (Figure 3b,c). Concerning the different ratios of the two fractions in the experiments (33:67) vs theory (45:55), it should be noted that the energy difference between two ensembles of nitroxides showing redistributed H-bonds is only 0.3 kcal/mol (according to Boltzmann population analysis), which could explain why the freezing of the sample could lead to minor modification of the ratio observed. Despite the existing deviations between the experimental and calculated absolute values of the parameters, the trends of the changes of the *g*_*xx*_ region and of the relative populations of the two dominant H-bonded states could be satisfactorily reproduced, indicating the robustness of the theoretical approach. Therefore, the smaller experimental peak at lower field was assigned to a population of HMIH^+^ with 1 H-bond and the peak with higher intensity at higher field was assigned to a population with 2 H-bonds.

**Figure 3:**
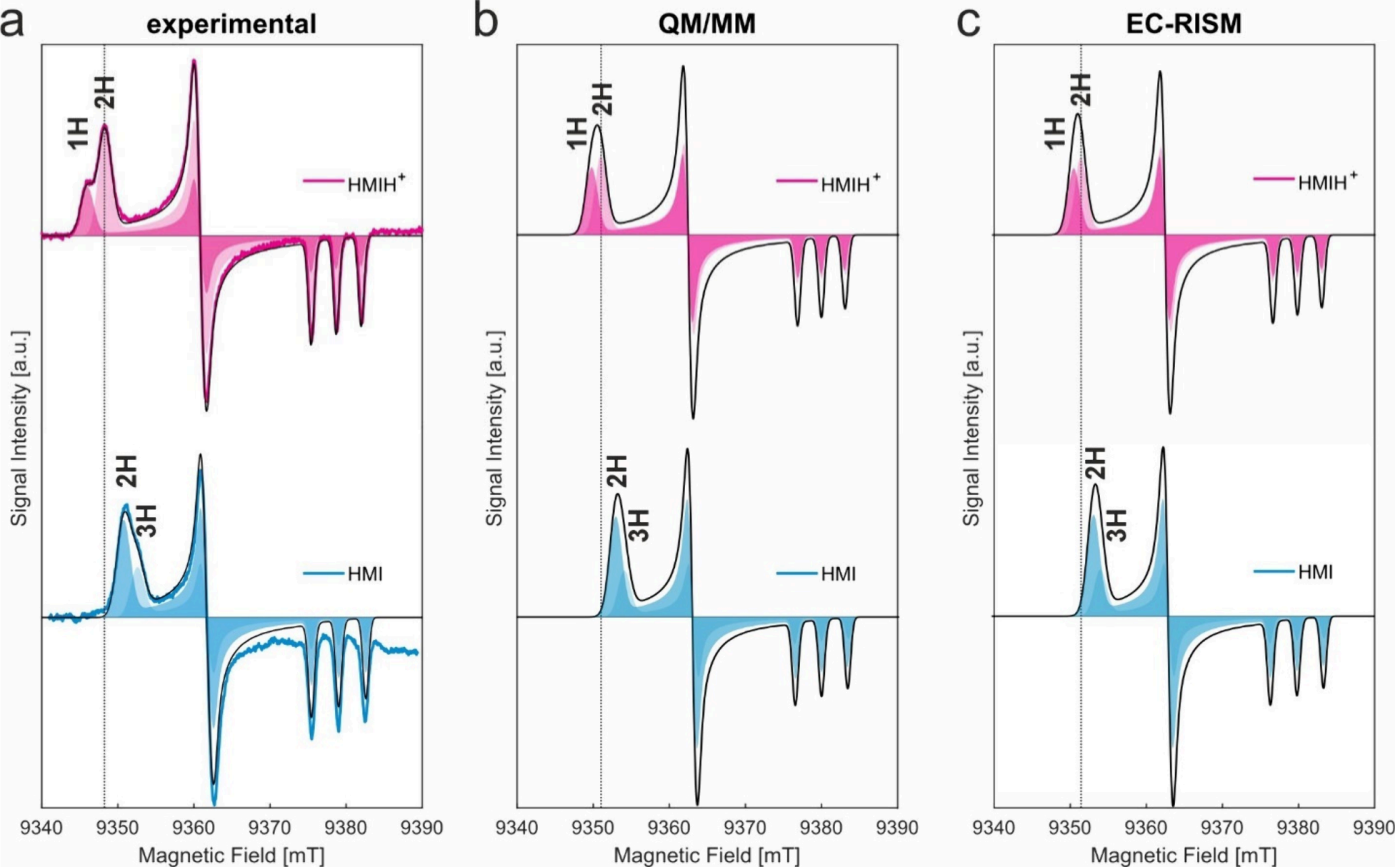
**Low-temperature J-band CW spectra of HMIH+ and HMI.** a) Experimental (colored thick lines) J-band EPR spectra (100 K) of HMI and HMIH^+^ and two-component EasySpin simulations (black thin lines) obtained using the parameters shown in Table 2 (for HMIH^+^) and from^16^ (for HMI) with the same inhomogeneous Gaussian line broadening (defined as *alw* in^16^). The two spectral components considered in the simulations are highlighted as shaded areas of different transparency, respectively. The two spectral components were assigned to populations with different H-bonds, depicted on the spectra. b,c) Simulated J-band EPR spectra using the parameters shown in Table 2 for QM/MM and EC-RISM, respectively. The two spectral components considered in the simulations are highlighted as shaded areas of different transparency, respectively. The weighted sum spectrum is shown in black. Dotted vertical lines corresponding to the lowest *g* value (2H-bonds) for HMIH^+^ (experiment or theory) are added to guide the eye and facilitate comparison between experimental and calculated trends. Parameters for HMI were partly taken from^16^ (see Table 4 therein, “exp-sim” and “solv-set1000-QM/MM”; Table S2, “revPBE0 level”) and recomputed for the two and three H-bond sub-ensembles stemming from the same snapshot set by EC-RISM.

**Table 2:** Parameters Used to Fit the Two Components (“Comp1”, “Comp2”) of the Low-Temperature Experimental Spectra Presented in Figure 3 of HMIH^+^ and HMI (from^16^) Compared to the Theoretical Parameters for the Two Dominant Calculated Components (“TComp1”, “TComp2”)^a^

		**Experimental (100 K)**		**QM/MM**		**EC-RISM** ^b^	
		*Comp1*	*Comp2*	*TComp1* ^a^	*TComp2* ^a^	*TComp1* ^a^	*TComp2* ^a^
**HMIH** ^**+**^	# H-bonds	1^c^	2^c^	1	2	1	2
	weights	0.33	0.67	0.45	0.55	0.45	0.55
	*g*_*xx*_	2.00937	2.00887	2.00855	2.00829	2.00837	2.00817
	(*g*_*xx*_-*g*_*zz*_)/10^–5^	700	650	643	618	625	605
	*g*_*yy*_	2.00617		2.00585	2.00581	2.00582	2.00577
	*g*_*zz*_	2.00237		2.00212	2.00211	2.00212	2.00212
	*A*_*xx*_ [MHz]	13.0		6.0	6.6	6.4	7.0
	*A*_*yy*_ [MHz]	13.0		6.4	6.9	6.7	7.2
	*A*_*zz*_ [MHz]	92.2		85.2	88.9	87.9	90.8
**HMI**	# H-bonds	2^c^	3^c^	2	3	2	3
	weights	0.67	0.33	0.69	0.31	0.69	0.31
	*g*_*xx*_	2.00834	2.00795	2.00787	2.00763	2.00778	2.0760
	(*g*_*xx*_ – *g*_*zz*_)/10^–5^	604	565	575	552	586	549
	*g*_*yy*_	2.00598		2.00572	2.00568	2.00570	2.00567
	*g*_*zz*_	2.00230		2.00212	2.00211	2.00212	2.00211
	*A*_*xx*_ [MHz]	14.0		7.3	7.4	7.5	7.8
	*A*_*yy*_ [MHz]	14.0		7.6	7.7	7.4	7.8
	*A*_*zz*_ [MHz]	100.0		95.3	98.2	96.5	98.7

a To validate our analysis on HMIH^+^, a multifrequency approach has been used (Figure S1). The error estimations on the chosen parameters are ±1 MHz for *A* values and ±10^–5^ for *g* values (see Figure S2), in analogy to our work on the unprotonated HMI.

b To better compare with the experimental spectra simulated with two components, we considered and renormalized the weights for only the two major theoretical populations (namely one and two H-bonds) that contribute to 89% of the cases for HMIH+ and the two and three H-bond sub-ensembles for HMI that contribute to 87% of the cases.^16^

c Values for HMI were recomputed based on the “solv-set1000-QM/MM” snapshot set from^15,16^ using ORCA5.0.3, which results in small differences to data previously derived.^15^

d Number of H-bonds assigned based on the calculated values.

All parameters derived by the two-component simulations of the experimental J-band spectra of HMI and HMIH^+^ with Easyspin are presented in Table 2, together with theoretically predicted values corresponding to one to two (HMIH^+^) and two to three (HMI) H-bonds, respectively.

The analysis of the low-temperature J-band spectra aided the interpretation of the components resolved in frozen aqueous solution and provided good control for the robustness of the calculated parameters. In a liquid solution at ambient temperature, the EPR spectra will only reflect the H-bond population-weighted average of the *g* and *A* tensorial quantities of HMI and HMIH^+^ at that temperature. All different H-bonded cases will therefore be encoded in the isotropic *g* and *A* parameters. However, we obtained slightly different values (Table 3, experimental data) by calculating the isotropic parameters from the experimental J-band spectra at 100 K or by direct determination from CW X-band EPR spectra at 298 K. The *g* ^*iso*^ values were only marginally different, however the *A* ^iso^ values differed by about 2 MHz, with *A* ^iso, 100 K^ < *A* ^iso, 298 K^ for both HMI and HMIH^+^. The temperature dependence of *A* ^iso^ has been identified and studied for 5- and 6-membered ring nitroxide radicals (see for example^29,30^ ). For 5-membered ring nitroxides (as for HMI) the *A* ^iso^ values was found to increase with temperature, with a positive slope d*A* ^iso^(*T*)/d*T* ^31^ in agreement with the changes observed in this study.

**Table 3:** *A* ^iso^ and *g* ^iso^ Values for HMI and HMIH^+^ Experimentally Obtained at 298 and 100 K Compared to the Quantities Calculated with VD, SSS, EC-RISM, and QM/MM^a^

	HMI (^a^)		HMIH^+^		HMI-HMIH^+^	
Method	*A*^iso^ [MHz]	*g* ^iso^	*A*^iso^ [MHz]	*g* ^iso^	Δ*A* ^iso^ [MHz]	Δ*g* ^iso^
**Exp. (298 K)**	**44.9**	**2.00550**	**41.2**	**2.00575**	**3.7**	**–0.00025**
**Exp. (100 K)**	**42.7**	**2.00550**	**39.4**	**2.00586**	**3.3**	**–0.00036**
**VD**	31.2(2)	2.00574(2)	27.8(2)	2.00589(1)	3.4(2)	–0.00015(2)
**SSS**	36.1(2)	2.00527(1)	31.8(2)	2.00554(1)	4.3(2)	–0.00027(1)
SSS-VD	4.9(2)	–0.00047(2)	4.0(2)	–0.00035(1)	0.9(3)	–0.00016(2)
SSS, 1 H-bond	34.4(5)	2.00539(1)	31.0(3)	2.00559(1)	3.3(5)	–0.00020(2)
SSS, 2 H-bonds	36.0(2)	2.00528(1)	32.5(3)	2.00550(1)	3.5(3)	–0.00022(1)
SSS, 3 H-bonds	37.1(3)	2.00519(1)	33.2(6)	2.00544(2)	3.9(7)	–0.00025(2)
⟨ΔH-bonds⟩_SSS_	-	-	-	-	3.6(3)	–0.00022(1)
⟨ΔH-bonds⟩_SSS_/ΔSSS	-	-	-	-	83%	81%
**QM/MM**	36.8(2)	2.00523(1)	33.4(2)	2.00546(1)	3.4(2)	–0.00023(1)
QM/MM-VD	5.6(2)	–0.00052(2)	5.6(2)	–0.00043(2)	0.0(3)	–0.00008(2)
QM/MM, 1 H-bond	34.9(5)	2.00536(1)	32.5(3)	2.00551(1)	2.3(5)	–0.00015(2)
QM/MM, 2 H-bonds	36.8(2)	2.00524(1)	34.1(3)	2.00541(1)	2.6(3)	–0.00017(1)
QM/MM, 3 H-bonds	37.8(3)	2.00514(1)	34.7(6)	2.00534(2)	3.0(6)	–0.00020(2)
⟨ΔH-bonds⟩_QM/MM_	-	-	-	-	2.7(3)	–0.00017(1)
⟨ΔH-bonds⟩_QM/MM_/ ΔQM/MM	-	-	-	-	78%	75%
**EC-RISM**	37.3(2)	2.00520(1)	34.4(2)	2.00540(1)	2.9(2)	–0.00020(1)
EC-RISM-VD	6.0(2)	–0.00055(2)	6.6(2)	–0.00049(2)	–0.5(3)	–0.00005(2)
EC-RISM, 1 H-bond	35.6(5)	2.00531(1)	33.7(3)	2.00544(1)	2.0(5)	–0.00013(2)
EC-RISM, 2 H-bonds	37.3(2)	2.00520(1)	35.0(3)	2.00536(1)	2.3(3)	–0.00015(1)
EC-RISM, 3 H-bonds	38.0(3)	2.00513(1)	35.6(6)	2.00529(2)	2.4(6)	–0.00016(2)
⟨ΔH-bonds⟩_EC‑RISM_	-	-	-	-	2.2(3)	–0.00015(1)
⟨ΔH-bonds⟩_EC‑RISM_/ ΔEC-RISM	-	-	-	-	77%	75%

a In bold: population-weighted averages over all H-bond sub-ensembles (0, 1, 2, 3, >3 H-bonds, bold) for the different methods, followed by difference to VD results, population-independent individual values per H-bond sub-ensemble, and (for Δ*A* ^iso^ and Δ*g* ^iso^ only) population-independent averages over all H-bond sub-ensembles. A graphical illustration of the EC-RISM and QM/MM parameters for each H-bond sub-ensemble is shown in Fig. 4. For Δ*A* ^iso^ and Δ*g* ^iso^, the exact values were taken for computing the difference and rounded afterwards. Statistical uncertainties were calculated as standard errors under the assumption of a normal distribution and statistically uncorrelated snapshots. Uncertainties of differences were estimated by error propagation.

b Values for HMI were recomputed based on the “solv-set1000-QM/MM” snapshot set from^15,16^ using ORCA 5.0.3, which results in small differences compared to the data in^15^.

Because here all AIMD simulations have been performed at 300 K, we find it more accurate and instructive to address the comparison between the experimental isotropic quantities detected at room temperature (*A* ^iso, 298 K^ and *g* ^iso, 298 K^, Table 3, Figure 4) and the same quantities calculated for the complete ensemble of H-bonds at 300 K (i.e., not only for the two dominantly populated H-bond patterns per species). The advantage of the theoretical analysis is that we can compare the output of different methods with the quantities calculated for vertically desolvated (VD) HMI and HMIH^+^, stratified over individual H-bonding states, to disentangle the contributions of the electronic structure changes upon protonation and individual H-bonding state population changes (Table 3).

**Figure 4:**
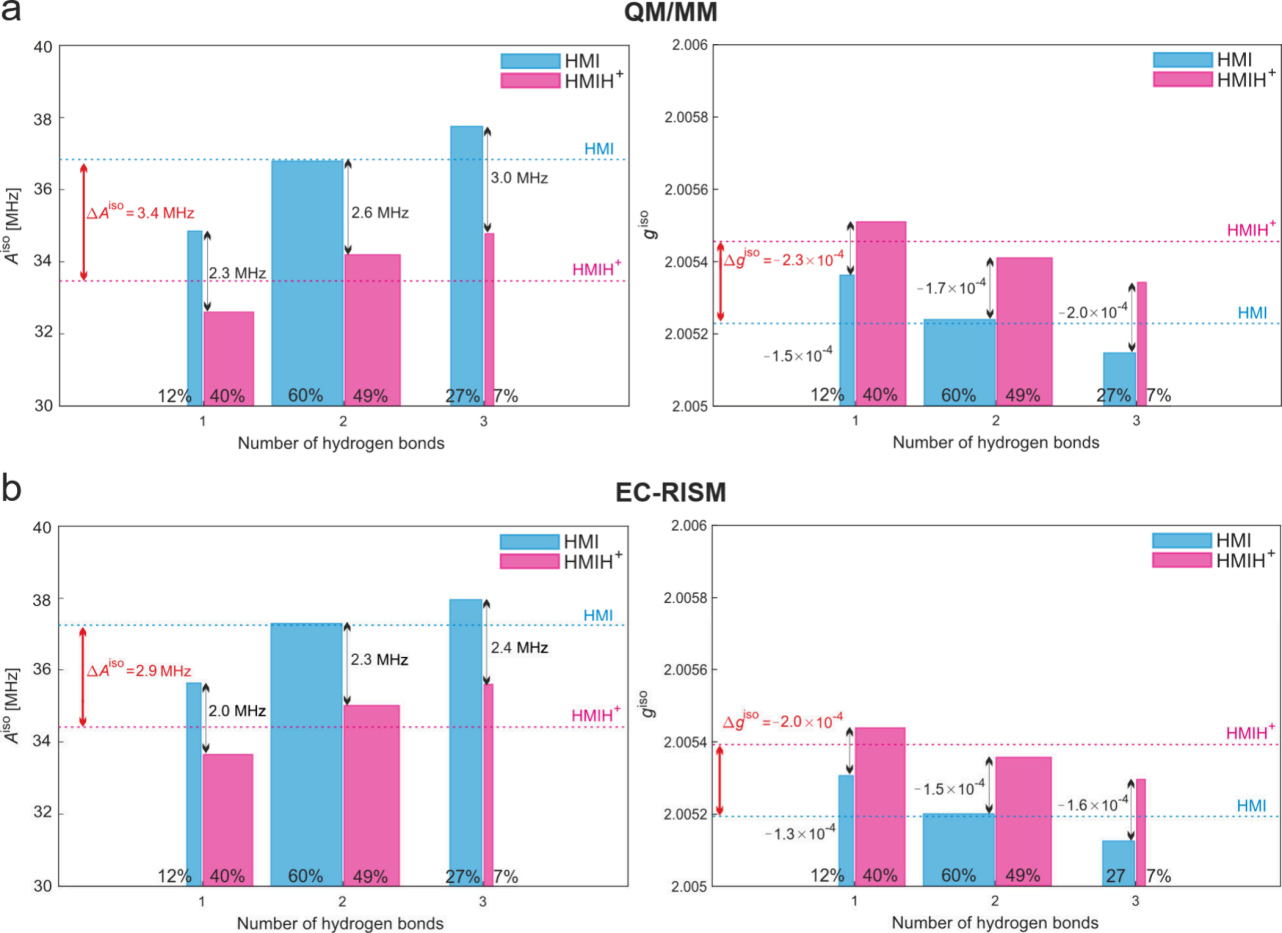
**Comparison of the computed *A* ^iso^ (left panels) and *g* ^iso^ (right panels) EPR parameters for HMI (blue) and HMIH^**+**^ (magenta) with a specific number of H-bonds.** For clarity, only the fractions with 1, 2, or 3 H-bonds covering 95.6% and 99% of the solvated radicals for HMI and HMIH^+^, respectively, are shown. The omitted small number of structures (<5% for HMI, <1% for HMIH^+^) corresponding to zero and more than three H-bonds are characterized by high statistical uncertainty. The parameters were calculated using QM/MM (a) and EC-RISM (b). The width of each bar corresponds to the population of the specific sub-ensemble annotated by the number of H-bonds. Horizontal dashed lines show the final average values. Differences between sub-ensembles are shown with black arrows, while the difference between the full ensembles is shown with a red arrow, where the solvation contribution is highlighted by a dashed line.

The results summarized in Table 3 provide an overview of absolute and relative contributions of solvation to *A* ^iso^ and *g* ^iso^, depending on the methodology (SSS, QM/MM, and EC-RISM with respect to VD). We first note that SSS alone already captures a large fraction of the solvation effect (with reference to the solvation-induced structural perturbation captured by the VD reference ensemble) on both EPR parameters. Further polarization from background charges adds a smaller increment (see lines “[SSS|QM/MM|EC-RISM]-VD”), which is slightly more relevant for HMIH^+^ than for HMI, from 4.9 MHz (SSS) to 6.1 MHz (EC-RISM) for *A* ^iso^ of HMI and from 4.0 to 6.6 MHz for *A* ^iso^ of HMIH^+^. In contrast, *g* ^iso^ appears to be less influenced by background polarization beyond the SSS, indicating a more locally confined solvation effect. This is also corroborated by the closer correspondence between QM/MM and EC-RISM for *g* ^iso^.

Additionally, the relative protonation effect based on the differences between HMIH^+^ and HMI has to be considered method-specifically from the point of view of individual H-bond contributions. Even though the electronic structure effect captured by the VD ensemble seemingly accounts for the total relative change of both *A* ^iso^ and *g* ^iso^ upon protonation, including solvation by using the SSS, EC-RISM, or QM/MM approaches clearly show strong solvation effects on absolute numbers. Further dissection by analyzing individual H-bond sub-ensembles will provide quantitative insight into the solvation effect on Δ*A* ^iso^ and Δ*g* ^iso^ since, as shown in Figure 2 and Table 1, their populations change strongly upon protonation.

A compact summary and illustration of individual H-bonded populations (in terms of one, two, or three H-bonds) on *A* ^iso^ and *g* ^iso^ values and their differences between protonated and neutral forms is provided in Figure 4 (data from Table 3). The differences are expected to be reliable on the DFT level unlike absolute values, as discussed in our previous work.^15^ Both Table 3 and Figure 4 show a clear dependence for *A* ^iso^ and *g* ^iso^ on the number of H-bonds for both HMI and HMIH^+^. *A* ^iso^ systematically increases with an increasing number of H-bonds while *g* ^iso^ decreases. This is in contrast to Δ*A* ^iso^ and Δ*g* ^iso^ that show almost no dependence on the number of H-bonds for all solvation models (SSS, QM/MM, and EC-RISM). It is noteworthy, and at first sight counterintuitive, that the overall changes highlighted in bold in Table 3 and in red in Figure 4 are *larger* for Δ*A* ^iso^ and *smaller* for Δ*g* ^iso^ (and also *larger* absolutes as Δ*g* ^iso^ is negative) than each individual Δ*A* ^iso^ and Δ*g* ^iso^ contribution per distinct H-bond ensemble. To explain this observation, Table 3 shows the average Δ*A* ^iso^ and Δ*g* ^iso^ for all three H-bond sub-ensembles. For example, individual H-bonding states yield a difference in *A* ^iso^ of 2.2 ± 0.2 MHz for EC-RISM (denoted by ⟨ΔH-bonds⟩_EC‑RISM_) and 2.7 ± 0.2 MHz for QM/MM (⟨ΔH-bonds⟩_QM/MM_). If the population of H-bond ensembles remained constant between HMI and HMIH^+^ these values would match the total Δ*A* ^iso^ and Δ*g* ^iso^ change for EC-RISM or QM/MM. However, this is not the case as the average over all H-bond sub-ensembles for all methods and both parameters, Δ*A* ^iso^ and Δ*g* ^iso^, consistently yields only ca. 80% of the total differences (denoted by ⟨ΔH-bonds⟩_SSS|EC‑RISM|QM/MM_/Δ­(SSS|EC-RISM|QM/MM).

How can this unexpected behavior be understood? Figure 4 with data from Tables 1 and 3 illustrates this point as well as the underlying reason: Populations shift from dominance of two and three H-bonds for HMI with the highest *A* ^iso^ and lowest *g* ^iso^ values upon protonation to dominantly populated one and two H-bonds for HMIH^+^ with the lowest *A* ^iso^ and highest *g* ^iso^ numbers. Consequently, the discrepancy between total and sub-ensemble-specific averages of both parameters is the result of *H-bond population modulations upon protonation* of HMI overlaying an intrinsically essentially constant difference within each individual H-bonding state.

The important finding and the underlying mechanistic picture are the following: As the ca. 80% contribution per H-bond (*before* weighting by populations) to the total average is consistent among all methods, and is found for both Δ*A* ^iso^ and Δ*g* ^iso^, we attribute this fraction to an *electronic structure effect* exhibited by a certain H-bond pattern (be it one, two or three H-bonds), that is consistent among all solvation models studied and valid for both EPR parameters. The remaining 20% can directly be assigned to the change in population between H-bond sub-ensembles or, in other words, traced back to a *solvation effect* of protonated versus neutral HMI in water.

It is noteworthy that the choice of the background solvation approximation beyond the SSS has an impact, especially on *A* ^iso^. The largest discrepancy of total averages between EC-RISM and QM/MM is 1 MHz (34.4 vs 33.4 MHz, respectively) for HMIH^+^ where the background solvation around the protonated nitrogen seems especially important due to longer-ranged solvent polarization expected from the presence of a net charge. In this case, consideration of finite-size artifacts arising from a small simulation cell is important, which are essentially eliminated by the much larger 3D grid (120^3^ point corresponding to a 60^3^ Å^3^ box) encompassing background charges in EC-RISM compared to QM/MM. To quantify such a finite-size effect, exemplary analyses were carried out for a representative set of snapshots (9 and 14 for HMI and HMIH^+^, respectively out of a total of 1000) with a reduced EC-RISM grid size of 46^3^ grid points corresponding to a 23^3^ Å^3^ box matching the QM/MM region. Taking the snapshots as an independent sample, *A* ^iso^ values of 32.9 ± 1.6 MHz (HMIH^+^) and 36.3 ± 0.9 MHz (HMI) were obtained. Compared to the EC-RISM reference, a smaller box introduces differences of −1.5 ± 1.6 MHz (HMIH^+^) and −0.9 ± 0.9 MHz (HMI), representing the upper limit of the standard error. Alternatively, average differences and associated standard errors can be estimated by computing first the per-snapshot difference to the corresponding EC-RISM reference frame before averaging. In this case, the smaller box exhibits a difference of −0.4 ± 0.2 MHz for HMIH^+^ and −0.3 ± 0.2 MHz for HMI compared to full EC-RISM. Both analyses are in line with the difference between QM/MM and EC-RISM of −1.0 ± 0.2 MHz for HMIH^+^ and −0.5 ± 0.2 MHz for HMI. Overall, our detailed analyses of the systematic finite-size errors validate our computational approach and make clear that these errors do not impact our conclusions as to the impact of protonation changes of HMI in water as seen by EPR.

In conclusion, we brought to the next level our previous efforts^15,16^ to combine accurate experiments and state-of-the art computational methods by investigating the molecular origin of the pH-dependency of *A* ^iso^ and *g* ^iso^ for the HMI spin probe. Extending the database obtained for neutral HMI,^15,16^ AIMD simulations on HMIH^+^ in aqueous solution revealed the populations of H-bonding states with predominantly one and two H-bonds around the oxygen site of the NO group, very different from the neutral HMI, which is characterized by having mostly a two and three H-bonding pattern of this group. Subjecting the AIMD-generated ensemble and its H-bond pattern sub-ensembles to EC-RISM and QM/MM-based calculations of the electronic structure yielded spectral predictions that closely matched experimental evidence. Comparing the experimental EPR spectra with the theoretical data obtained on HMIH^+^ with those previously obtained on the neutral form of HMI, we found good agreement in the shift of the major populations of H-bonds as well as of the isotropic *A* and *g* parameters.

This validation step allowed us to faithfully analyze and dissect the interplay of electronic structure perturbation upon protonation in a specific H-bonding state and protonation-induced modulation of H-bonding state populations, yielding three key insights regarding differences between HMI and HMIH^+^ as follows: (1) The H-bonding structures of HMI and HMIH^+^ around the nitroxy group are not fundamentally different; only the populations of sub-ensembles belonging to different H-bond numbers change, whereby protonation of the HMI molecule leads to a reduction of the average H-bond number. Every added H-bonded water molecule brings along an additive perturbation to both *A* ^iso^ and *g* ^iso^ that is very similar for HMI and HMIH^+^ as derived from the near-constant “HMI minus HMIH^+^” differences for the averages of *A* ^iso^ and *g* ^iso^ belonging to each H-bonding state. (2) The dominant factor accounting for roughly 80% of the total change of both *A* ^iso^ and *g* ^iso^ upon protonation is the *perturbation of the electronic structure* within a specific H-bonding state. This was concluded from a comparison of sub-ensembles with a given number of hydrogen bonds for various solvation models to the total average values for Δ*A* ^iso^ and Δ*g* ^iso^. (3) Hence, the remaining contribution of about 20% to the overall protonation effect on EPR parameters results from the modulation of the subpopulations of H-bonding states and, thus, from different solvation changes upon protonating HMI in water

This comprehensive analysis that rigorously dissects the interplay of (intramolecular HMI) electronic effects and (intermolecular HMI–water) solvation effects upon protonation provides the key ingredients for rationalizing the pH response of the HMI spin probe in solution. As different H-bond patterns exhibit different responses of EPR parameters, it is conceivable to use HMI as a probe for sensing environmental changes correlated with different H-bond populations (for instance, local water depletion in mixed solvents as a function of pH), or H-bonding networks in biomolecular complexes. If needed, the molecular-level understanding will then be contributed by calculations such as those introduced here but scaled-up to larger systems, e.g., by QM/MM molecular dynamics simulations instead of full AIMD followed by quantum chemical analysis along the lines developed and validated in this and our previous works.

## Methods

Methods are described in the Supporting Information and in the associated content (see Data Availability Statement).

## Supplementary Material



## Data Availability

Raw computational data (snapshots from AIMD simulations of HMI and HMIH^+^ in water with corresponding calculated EPR parameters (*g*-tensor and ^14^N hyperfine coupling constants *A*) is provided in machine-readable format under https://doi.org/10.17877/RESOLV-2025-M7UFWH5H.

## Notes

Sample preparation; low-temperature CW EPR: experimental parameters; room-temperature CW EPR: experimental parameters; EPR data analysis; error estimation on the different components of the hyperfine tensor; ab initio molecular dynamics of HMI and HMIH^+^ in water; DFT calculations of EPR parameters (PDF)

## ¶

Department of Chemistry and Applied Biosciences, ETH Zürich, Vladimir-Prelog-Weg 2, 8093 Zurich, Switzerland. (L.G.)

## ■

Department of Biological Sciences and Bioengineering, Indian Institute of Technology Kanpur, Kanpur 208016, India. (B.S.)
