# Supplementary material for: Origin of the pH Dependency of EPR Parameters: The Case of a Protonatable Nitroxide in Aqueous Solution
Source: J Phys Chem Lett. 2025 Aug 1;16(32):8141–9. doi: 10.1021/acs.jpclett.5c01053 (PMC12359196; doi:10.1021/acs.jpclett.5c01053)
Supplement: Supplementary file 1 [file jz5c01053_si_001.pdf]

Origin of the pH dependency of EPR parameters: The case of a protonatable nitroxide in aqueous solution

Laura Galazzo[1,2,#], Stefan Maste[3,#], Bikramjit Sharma[4,5#], Van Anh Tran[6], Tim Pongratz[3], Markus Teucher[7], Dominik Marx[4,], Frank Neese[6,*], Stefan M Kast[3,*], Enrica Bordignon[1,*]*

[1] *Department of Physical Chemistry, University of Geneva, 30 Quai Ernest Ansermet, 1211 Geneva, Switzerland*

[2] *current address: Department of Chemistry and Applied Biosciences, ETH Zürich, Vladimir-Prelog-Weg 2, 8093 Zurich, Switzerland*

[3] *Fakultät für Chemie und Chemische Biologie, Technische Universität Dortmund, 44227 Dortmund, Germany*

[4] *Lehrstuhl für Theoretische Chemie, Ruhr-Universität Bochum, 44780 Bochum, Germany*

[5] *current address: Department of Biological Sciences and Bioengineering, Indian Institute of Technology Kanpur 208016, India*

[6] *Max-Planck-Institut für Kohlenforschung, 45470 Mülheim an der Ruhr, Germany*

[7] *Max-Planck-Institut für Chemische Energiekonversion, 45470 Mülheim an der Ruhr, Germany*

Supplementary Information

Sample preparation

The HMI spin probe (2,2,3,4,5,5-hexamethylimidazolidin-1-oxyl, $C_9H_{19}N_2O$) was synthesized as described in Ref.[1-4] For the EPR experiments, solutions containing 500 μM HMI were prepared in ultra-pure milli-Q water.

HCl at a final concentration of 10^{-2} M was added (pH=2 measured with a FiveEasy Plus FP20 pH-meter from Mettler Toledo) to ensure that the protonated HMI form ($HMIH^+$) was predominant (the pK_A of HMI is about 4.5 at ambient temperature[5]). To prepare the samples for the low-temperature measurements, 10% (v/v) d-glycerol was added as a cryoprotectant to the samples, which were inserted in the EPR tubes and shock-frozen in liquid nitrogen. For the room temperature samples, no glycerol was added.

For the sample at pH=10 shown in Figure 1, NaOH at a final concentration of 10^{-4} M was added, while the one at pH=4.5 was prepared by dilution of the sample at pH=2 using a solution of HMI 500 μM in ultra-pure milli-Q water until the desired pH was reached.

Low-temperature CW EPR: experimental parameters

The J-band EPR (262.8 GHz/9.38 T) data were acquired with a Bruker ELEXSYS 780 spectrometer equipped with a non-resonant sample insert. The spectra were acquired at 100 K, with 1.5 mW microwave power, a modulation amplitude of 1 mT, a conversion time of 800 ms, a field sweep of 50 mT, and 2024 points. The modulation amplitude for lock-in detection of the CW EPR spectra was adjusted for each frequency band to achieve optimum sensitivity and, at the same time, guarantee optimum resolution of the spectral features.

CW X-band EPR (9.8 GHz/0.35 T) experiments were performed using a Bruker ELEXSYS E580 (Rheinstetten, Germany) spectrometer in conjunction with a Bruker MD-5 resonator. The spectra were acquired at 100 K with 0.013 mW microwave power, a modulation amplitude of 0.1 mT, a conversion time of 40 ms, a field sweep of 20 mT, and 1024 points.

CW Q-band EPR (33.7 GHz/1.2 T) experiments were performed using the same spectrometer with a custom-built Q-band accessory and a Bruker CW resonator. The spectra were acquired at 100 K, with 4 mW microwave power, a modulation amplitude of 0.5 mT, a conversion time of 40 ms, a field sweep of 20 mT, and 1024 points. 32 spectra were averaged to optimize the signal-to-noise ratio.

For the CW W-band EPR (94 GHz/3.35 T) measurements, a modified Bruker ELEXSYS II E680 spectrometer. The spectra were acquired at 100 K with $5.6*10^{-5}$ mW microwave power, a modulation amplitude of 0.3 mT, a conversion time of 81.92 ms, a field sweep of 60 mT, and 8192 points.

The CW EPR spectra were post-processed using a frequency- and spectrometer-specific magnetic field calibration, as described in Ref.[6]

Room temperature CW EPR: experimental parameters

X-Band CW EPR measurements were carried out on a Bruker ELEXSYS E580 X-band spectrometer equipped with a Bruker ER 4122HSQ super-high Q cavity. The temperature was kept constant at 295 K with a Bruker liquid nitrogen variable temperature unit. The samples were loaded in capillaries of 0.9 mm inner diameter, and the spectra were acquired with the following parameters: 9.2 GHz microwave frequency, 0.47 mW power, 100 G sweep width, 0.8 G modulation amplitude, 117.19 ms conversion time, 1024 points, and 1 scan.

EPR data analysis

The multi-frequency EPR spectra were post-processed using a frequency- and spectrometer-specific magnetic field calibration, as described in Ref.[6] Subsequent spectral simulations were carried out with the MATLAB routine "pepper" from EasySpin (version 5.2.33),[7] as also described in Ref.[6]

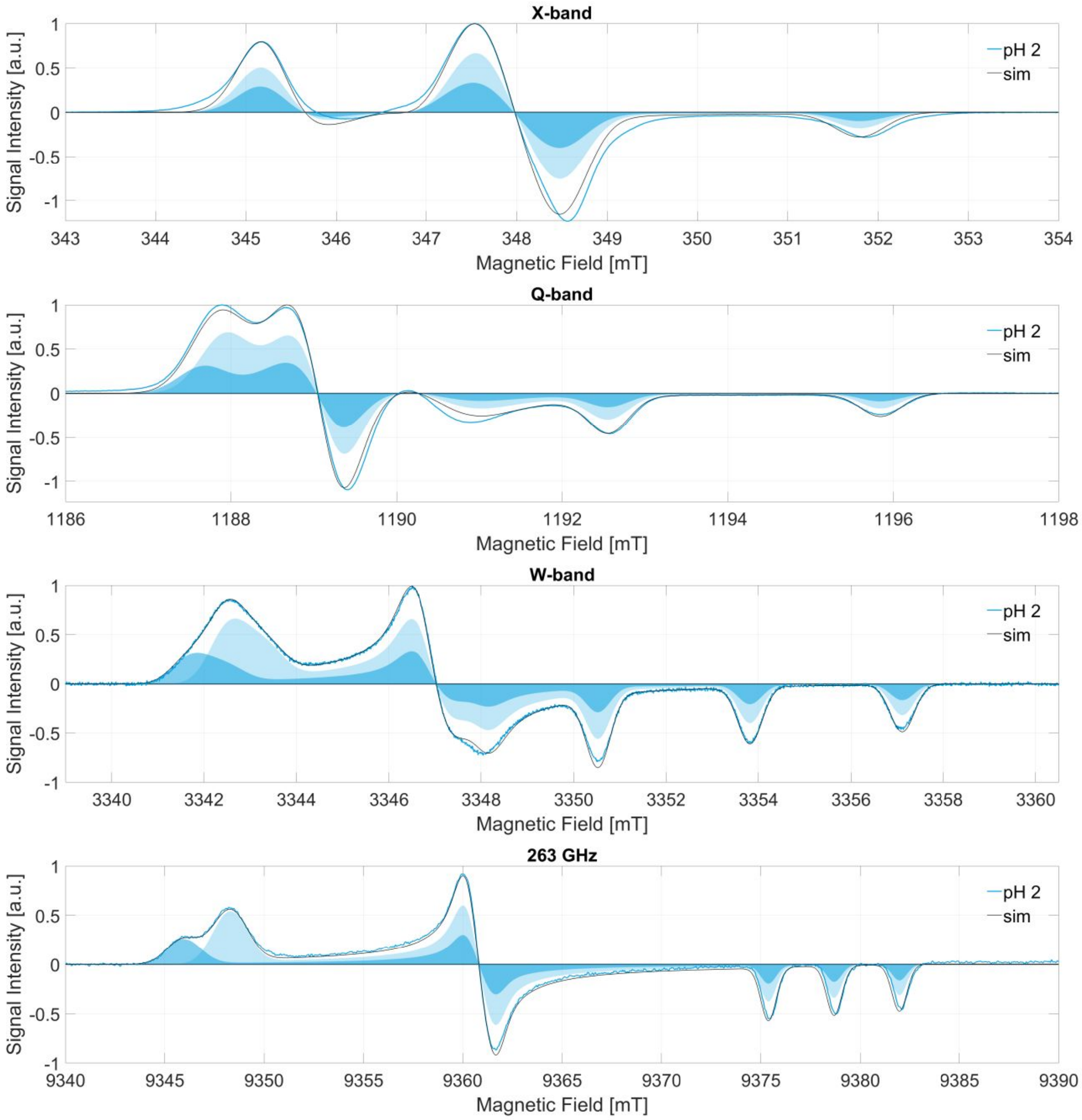

Figure S1: Multi-frequency analysis of the EPR spectra recorded at X, Q, W and J band using the Matlab routine EasySpin. The parameters used for the simulations are reported in the main text in Table 2. The shaded areas indicate the two different resolved components, as discussed in the main text.

Error estimation on the different components of the hyperfine tensor

To validate our analysis, we performed an error estimation on the different components of the hyperfine tensor, as these are fundamental parameters for the comparison of the isotropic values between experiments and theory.

In particular, we simulated the W-band EPR spectra fixing two components of the tensor and varying only one in steps of 1 MHz (left panels in Figure S2). Then, the standard deviation between simulated and experimental spectra was calculated and plotted against the corresponding hyperfine value (right panel in Figure S2). The value with the smallest standard deviation was then chosen for the final simulations. Thanks to this analysis, we estimated an error of ± 1MHz on the hyperfine values used for the simulations.

A similar approach has been used for the components of the *g* tensor (data not shown).

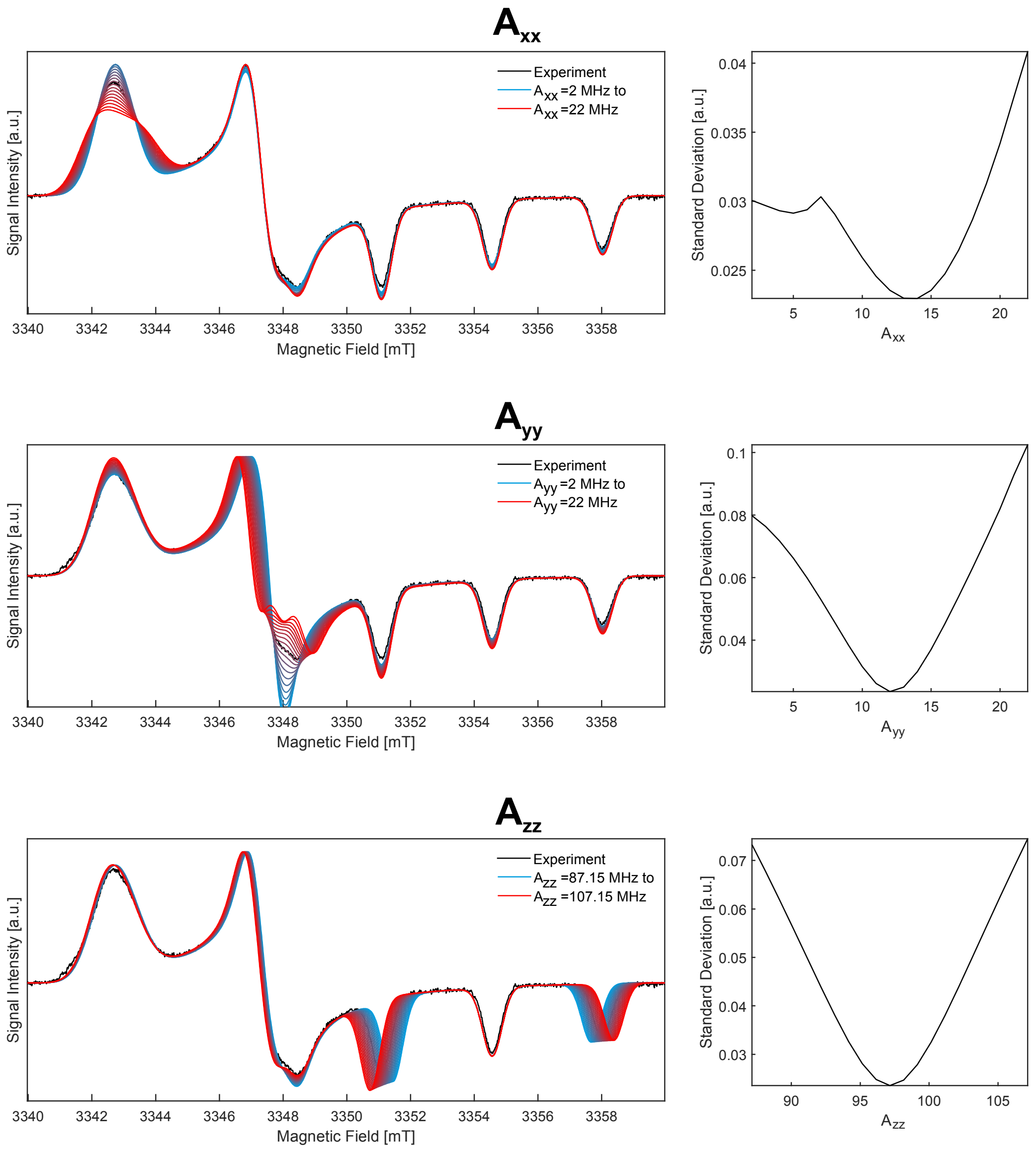

Figure S2: On the left: simulations of W-band EPR spectra with one parameter which is varied in steps of 1 MHz (the blue is the starting value and the red the end value as indicated in each legend). On the right: standard deviation between experimental and simulated spectrum for each *A* component tested.

Ab initio molecular dynamics of HMI and $HMIH^+$ in water

We have performed two separate ab initio molecular dynamics (AIMD) simulations of one neutral HMI (see Ref.[1]) and the protonated HMI ($HMIH^+$) molecules in cubic periodic simulation boxes containing 128 water molecules. The Born–Oppenheimer propagation[8] was employed to dynamically evolve the systems. For these AIMD simulations, the CP2K code[9-10] was used wherein the electronic structure was calculated by means of the QUICKSTEP[11] module. An atom-centered Gaussian basis set, namely TZV2P together with Goedecker–Teter–Hutter pseudopotentials[12-14] was used to represent the Kohn–Sham orbitals. A plane wave basis with a kinetic energy cutoff of 500 Ry was used to compute the total electron density. All the simulations were carried out using the spin-polarized hybrid revPBE0 density functional[15-16] together with the D3 dispersion correction[17]. The computation of the the Fock exchange terms of the revPBE0-D3 functional was accelerated by using the auxiliary density matrix method[18] with the cpFIT3 auxiliary basis. The simulations were conducted in the NVT ensemble using massive Nose–Hoover chain thermostatting[19] and a timestep of 0.5 fs was used to integrate the equations of motions. Both AIMD trajectories were generated for a total length of 206 ps. The first 6 ps was considered as the equilibration period and the remaining 200 ps was used to extract snapshots for quantum chemical calculations of the EPR observables. For both HMI and $HMIH^+$, snapshots after every 200 fs from the respective AIMD trajectories were extracted leading to a total of 1000 snapshots respectively, for subsequent DFT calculations of the EPR parameters.

DFT calculations of EPR parameters

The EPR calculations followed the previously benchmarked methodology.[1,6] A hierarchy of solvation methods was employed taking the same set of 1000 snapshots from AIMD simulations. For the vertically desolvated (VD) snapshots only the solute was used within the EPR calculations. For the explicit second solvation shell (SSS) water within 5.5 Å around the nitroxide-oxygen was included. These were augmented by QM/MM and EC-RISM solvation. For the QM/MM solvation approach water molecules in an outer sphere of the simulation cell from AIMD snapshots were represented as TIP3P point charges.[20] All molecular geometries together with QM/MM point charges are listed in the electronic supporting information.

The EC-RISM calculations were performed on a cubic grid with 120^3 points and a grid spacing of 0.5 Å. A limited number of EC-RISM calculations were also performed using only 46^3 points to analyse the grid size effect. The water solvent susceptibility was taken from earlier work[21] using a modified SPC/E model with a dielectric constant of 78.4 and a number density of 0.0333295 $Å^{-3}$. The LJ parameters were taken from GAFF force field version 1.4.[22] Auxiliary atom-centered point charges were calculated with the CHelpG scheme using Breneman–Wiberg radii[23] with a 0.3 Å grid spacing and a maximum distance of all atoms to any grid point of 2.8 Å. The convergence criteria were set to 10^{-6} for the maximum residual norm of direct correlation functions within 3D RISM calculations and to 0.01 kcal mol^{-1} for the maximum excess chemical potential difference between two consecutive EC-RISM cycles. Only in the last iteration, after convergence of the EC-RISM cycle, the EPR calculation was performed. The embedding point charges taken from the last EC-RISM iteration and used for EPR calculations are given in the electronic supporting information.

All EPR calculations were performed using ORCA 5.0.3.[24] This includes redoing previous calculations of HMI for methodological consistency. All EPR calculations were performed using again the revPBE0 density functional[15-16] together with the D3 dispersion correction[17] and the def2-TZVPP basis set[25] with decontracted s-functions. Example input files for EPR calculations are given in the electronic supporting information.

References

1. Sharma B.; Tran V.A.; Pongratz T.; Galazzo L.; Zhurko I.; Bordignon E.; Kast S. M.; Neese F.; Marx D. A Joint Venture of Ab Initio Molecular Dynamics, Coupled Cluster Electronic Structure Methods, and Liquid-State Theory to Compute Accurate Isotropic Hyperfine Constants of Nitroxide Probes in Water. J. Chem. Theory Comput. 2021, 17, 6366-6386.

2. Volodarskii L. B.; Reznikov V.; Kobrin V. Preparation and Properties of Imidazolinium Salts Containing a Nitroxyl Radical Center. J. Org. Chem. 1979, 15, 415-422.

3. Volodarskii L.; Reznikov V.; Kobrin V. Preparation and Properties of Imidazolinium Salts Containing a Nitroxyl Radical Center. Chem. Abstr. 1982, 91, 5158w.

4. Sevast'yanova T. K.; Volodarskii L. B. Preparation of stable iminoxyl radicals of 3-imidazolines. Bull. Russ. Acad. Sci.: Phys. 1972, 21, 2276-2278.

5. Margita K.; Voinov M. A.; Smirnov A. I. Effect of Solution Ionic Strength on the pK(a) of the Nitroxide pH EPR Probe 2,2,3,4,5,5-Hexamethylimidazolidin-1-oxyl. Cell Biochem. Biophys. 2017, 75, 185-193.

6. Tran V.A.; Teucher M.; Galazzo L.; Sharma B.; Pongratz T.; Kast S.M.; Marx D.; Bordignon E.; Schnegg A.; Neese F.; Dissecting the Molecular Origin of g-Tensor Heterogeneity and Strain in Nitroxide Radicals in Water: Electron Paramagnetic Resonance Experiment versus Theory. J. Phys. Chem. A. 2023, 127, 6447-6466.

7. Stoll S.; Schweiger A. EasySpin, a comprehensive software package for spectral simulation and analysis in EPR. J. Magn. Reson. 2006, 178, 42-55.

8. Marx, D.; Hutter, J. Ab Initio Molecular Dynamics: Basic Theory and Advanced Methods; Cambridge University Press, 2010.

9. https://www.cp2k.org/ (accessed June 16, 2025).

10. Hutter, J.; Iannuzzi, M.; Schiffmann, F.; VandeVondele, J. Cp2k: Atomistic Simulations of Condensed Matter Systems. Wiley Interdiscip. Rev.: Comput. Mol. Sci. 2014, 4, 15−25.

11. VandeVondele, J.; Krack, M.; Mohamed, F.; Parrinello, M.; Chassaing, T.; Hutter, J. Quickstep: Fast and Accurate Density Functional Calculations Using a Mixed Gaussian and Plane Waves Approach. Comput. Phys. Commun. 2005, 167, 103−128.

12. Goedecker, S.; Teter, M.; Hutter, J. Separable Dual-Space Gaussian Pseudopotentials. Phys. Rev. B: Condens. Matter Mater. Phys. 1996, 54, 1703.

13. Krack, M. Pseudopotentials for H to Kr Optimized for Gradient-Corrected Exchange-Correlation Functionals. Theor. Chem. Acc. 2005, 114, 145−152.

14. Hartwigsen, C.; Goedecker, S.; Hutter, J. Relativistic Separable Dual-Space Gaussian Pseudopotentials from H to Rn. Phys. Rev. B: Condens. Matter Mater. Phys. 1998, 58, 3641.

15. Adamo, C.; Barone, V. Toward reliable density functional methods without adjustable parameters: The PBE0 model. J. Chem. Phys. 1999, 110, 6158.

16. Zhang, Y.; Yang, W. Comment on "Generalized Gradient Approximation Made Simple". Phys. Rev. Lett. 1998, 80, 890.

17. Grimme, S.; Antony, J.; Ehrlich, S.; Krieg, H. A Consistent and Accurate Ab Initio Parametrization of Density Functional Dispersion Correction (DFT-D) for the 94 Elements H-Pu. J. Chem. Phys. 2010, 132, 154104.

18. Guidon, M.; Hutter, J.; VandeVondele, J. Auxiliary Density Matrix Methods for Hartree- Fock Exchange Calculations. J. Chem. Theory Comput. 2010, 6, 2348−2364.

19. Martyna, G. J.; Klein, M. L.; Tuckerman, M. Nose-Hoover Chains: The Canonical Ensemble via Continuous Dynamics. J. Chem. Phys. 1992, 97, 2635.

20. Jorgensen, W. L.; Chandrasekhar, J.; Madura, J. D.; Impey, R. W.; Klein, M. L. Comparison of simple potential functions for simulating liquid water. J. Chem. Phys. 1983, 79, 926–935.

21. Pongratz, T.; Kibies, P.; Eberlein, L.; Tielker, N.; Hölzl, C.; Imoto, S.; Beck Erlach, M.; Kurrmann, S.; Schummel, P. H.; Hofmann, M.; Reiser, O.; Winter, R.; Kremer, W.; Kalbitzer, H. R.; Marx, D.; Horinek, D.; Kast, S. M. Pressure-dependent electronic structure calculations using integral equation-based solvation models. Biophys. Chem. 2020, 257, 106258.

22. Wang, J.; Wolf, R. M.; Caldwell, J. W.; Kollman, P. A.; Case, D. A. Development and testing of a general amber force field. J. Comput. Chem. 2004, 25, 1157–1174.

23. Breneman, C. M.; Wiberg, K. B. Determining atom-centered monopoles from molecular electrostatic potentials. The need for high sampling density in formamide conformational analysis. J. Comput. Chem. 1990, 11, 361–373.

24. Neese, F.; Wennmohs, F.; Becker, U.; Riplinger, C. The ORCA quantum chemistry program package. J. Chem. Phys. 2020, 152, 224108.

25. Weigend, F.; Ahlrichs, R. Balanced basis sets of split valence, triple zeta valence and quadruple zeta valence quality for H to Rn: Design and assessment of accuracy. Phys. Chem. Chem. Phys. 2005, 7, 3297–3305.
